# A novel endoscopic capsule sponge-based technique called transendoscopic sampling for early detection and risk stratification in Barrett’s neoplasia

**DOI:** 10.1055/a-2769-7061

**Published:** 2025-12-12

**Authors:** Pim Stougie, Danique Wajon, Roos Pouw, Gem M. Kramer, Lucas C. Duits

**Affiliations:** 1 1209 Gastroenterology and Hepatology, Amsterdam UMC Locatie VUmc, Amsterdam, The Netherlands; 2 8124 Gastroenterology and Hepatology, UMC Utrecht, Utrecht, The Netherlands; 3 1209 Amsterdam Gastroenterology, Endocrinology and Metabolism, Amsterdam UMC, University of Amsterdam, Amsterdam UMC Location VUmc, Amsterdam, Netherlands

Effective surveillance of Barrett’s esophagus (BE) requires representative sampling of the metaplastic epithelium to enable the early detection of dysplasia or adenocarcinoma. However, the current gold standard of forceps biopsies is limited by the sampling error, interobserver variability in histopathological assessment, and poor integration with emerging molecular diagnostic strategies. Although novel widefield sampling devices have shown promise for more extensive coverage of the BE segment, their clinical adoption is often restricted by patient intolerance or inadequate sampling. As a result, obtaining truly representative tissue samples remains technically challenging.

We use a transendoscopic sampling (TES) technique ( Video 1 ) involving a dissolvable capsule sponge to enable comprehensive sampling during routine endoscopy. The capsule is introduced into the stomach alongside the endoscope using a biopsy forceps. Upon dissolution, it expands into a rough-textured sponge. While the endoscopist inspects and cleans the BE segment, the sponge is deployed and subsequently retrieved transorally, collecting cells from the entire BE without cross-contamination. The procedure results in visible abrasions across the entire BE segment, including targeted suspicious lesions.

Transendoscopic sampling (TES) using a dissolvable capsule sponge enables widefield sampling during endoscopy, leaving clear abrasions across Barrett’s segment, including a suspicious lesion, while yielding sufficient DNA for molecular analyses.Video 1

In a prospective series of 226 TES procedures, the technical success was achieved in 224/226 cases (99.1%). 2/226 procedures failed due to severe esophageal stenosis that prevented the passage of the capsule sponge. All 64 BE patients with histologically confirmed progression (high-grade dysplasia or esophageal adenocarcinoma) or endoscopically suspicious lesions underwent successful sampling. In 100% of the successfully retrieved samples (224/224), sufficient material was collected for DNA isolation.

Transendoscopic sampling using the capsule sponge is a safe, well-tolerated, and technically feasible procedure for widefield sampling of BE. It offers a scalable platform for integrating molecular biomarkers into routine surveillance and has the potential to complement, or even revolutionize, endoscopic surveillance in Barrett’s esophagus.

Endoscopy_UCTN_Code_TTT_1AO_2AC

## Notes

Endoscopy E-Videos https://eref.thieme.de/e-videos
*E-Videos* is an open access online section of the journal *Endoscopy* , reporting on interesting cases and new techniques in gastroenterological endoscopy. All papers include a high-quality video and are published with a Creative Commons CC-BY license. Endoscopy E-Videos qualify for HINARI discounts and waivers and eligibility is automatically checked during the submission process. We grant 100% waivers to articles whose corresponding authors are based in Group A countries and 50% waivers to those who are based in Group B countries as classified by Research4Life (see: https://www.research4life.org/access/eligibility/ ). This section has its own submission website at https://mc.manuscriptcentral.com/e-videos .

